# The ischiofemoral space of the hip is influenced by the frontal knee alignment

**DOI:** 10.1007/s00167-021-06589-6

**Published:** 2021-05-05

**Authors:** Sufian S. Ahmad, Vincent Kerber, Christian Konrads, Atesch Ateschrang, Michael T. Hirschmann, Ulrich Stöckle, Marc D. Ahrend

**Affiliations:** 1 BG Center for Trauma and Reconstructive Surgery, Eberhard-Karls University of Tübingen, Tübingen, Germany; 2 Center for Musculoskeletal Surgery, Charité - Universitätsmedizin Berlin, Berlin, Germany; 3 Evangelisches Stift St. Martin Gemeinschaftsklinikum Mittelrhein, Koblenz, Germany; 4 Department of Orthopaedic Surgery and Traumatology, Kantonsspital Baselland (Bruderholz, Liestal, Laufen), 4101 Bruderholz, Switzerland

**Keywords:** Knee malalignment, Valgus knee, Varus knee, Femoroacetabular impingement, FAI, Ischiofemoral space, Hip impingement, Hip conflict, Extra-articular hip impingment, Extra-articular hip impingment

## Abstract

**Purpose:**

The ischiofemoral distance (IFD), defined as the distance between the ischial tuberosity and the lesser trochanter of the femur, is gaining recognition as an extra-articular cause of hip pain. It is unknown whether the IFD is influenced by the frontal knee alignment. The aim of this study was to determine the influence of realignment surgery around the knee on the IFD. It was hypothesized that valgisation osteotomy around the knee is associated with reduction of the IFD.

**Methods:**

A consecutive series of 154 patients undergoing frontal realignment procedures around the knee in 2017 were included in this study. Long-leg standing radiographs were obtained before surgery and postoperatively. The IFD was measured between the ischium and the lesser trochanter at three different levels (proximal, middle and distal margins of the lesser trochanter parallel to the horizontal orientation of the pelvis) on standardized long-leg radiographs with the patient in upright standing position. The knee alignment was determined by measuring the hip knee ankle angle, mechanical lateral distal femur angle and the medial mechanical proximal tibia angle. Linear regression was performed to determine the influence of the change of frontal knee alignment on the IFD.

**Results:**

Linear regression showed a direct influence of the overall change in frontal knee alignment on the IFD of the hip, regardless of the site of the osteotomy (β-0.4, confidence-interval − 0.5 to − 0.3, *p* < 0.001). Valgisation osteotomy around the knee induced a significant reduction of the ipsilateral IFD (*p* < 0.001), while varisation osteotomy induced a significant increase (*p* < 0.001). The amount of ISD change was 0.4 mm per corresponding degree of change in frontal knee alignment.

**Conclusion:**

These findings are relevant to both the hip and knee surgeons when planning an osteotomy or arthroplasty procedure. Correction of a malalignment of the knee may resolve an ischiofemoral conflict in the hip. The concept deserves inclusion in the diagnostic workup of both the hip and knee joints.

**Level of evidence:**

IV.

## Introduction

Given the fact that the knee is connected to the hip by the femur and to the ankle by the tibia, it is reasonable to expect that the alignment of one joint is likely to exhibit an influence on adjacent joints [8, 15, 20]. The knowledge of such interactions is essential in the era of sub-specialization, where the hip and knee surgeons may not be familiar with the concepts of one another [12]. This would not only limit the likelihood of unwanted effects of an osteotomy on adjacent joints, but also widen the spectrum of treatment options.

This study will be dealing with the effect of surgical realignment of the knee on the distance between the ischial tuberosity of the pelvis and the lesser tuberosity of the femur, also known as the ischiofemoral distance [14]. The ischiofemoral distance is increasingly being recognized for its role in the pathogenesis of extra-articular hip pain [26]. The pathology has frequently been referred to as ischiofemoral impingement [2, 3, 22]. The reduced distance between the lesser trochanter and the ischium was shown to be associated with compression of the quadratus femoris muscle occupying the anatomic space [25]. High femoral antetorsion as well as a valgus morphology of the femoral neck have been found to be associated with this impingement entity [19, 22]. Several treatment options have been proposed including conservative management, CT-guided injection of the quadratus femoris muscle and advancement or resection of the lesser trochanter [7, 9]. The Bernese group proposed torsional correction osteotomy as the treatment option of choice [22]. Promising clinical results have been published in that regard [4, 13].

The association and relationship between the frontal alignment of the knee and the ischiofemoral space of the hip is unknown. The clinical relevance here may be seen in the need to address a problem around the knee in order to resolve a hip problem. This emphasizes the novelty aspect of the current study. The aim was therefore to examine the influence of correctional osteotomy around the knee on the ischiofemoral distance of the hip. Given that adduction of the femur is possibly necessary to maintain contact between the foot and the floor in a valgus knee, it was hypothesized that valgisation osteotomy around the knee is associated with reduction of the ischiofemoral distance.

## Materials and methods

**Fig. 1:**
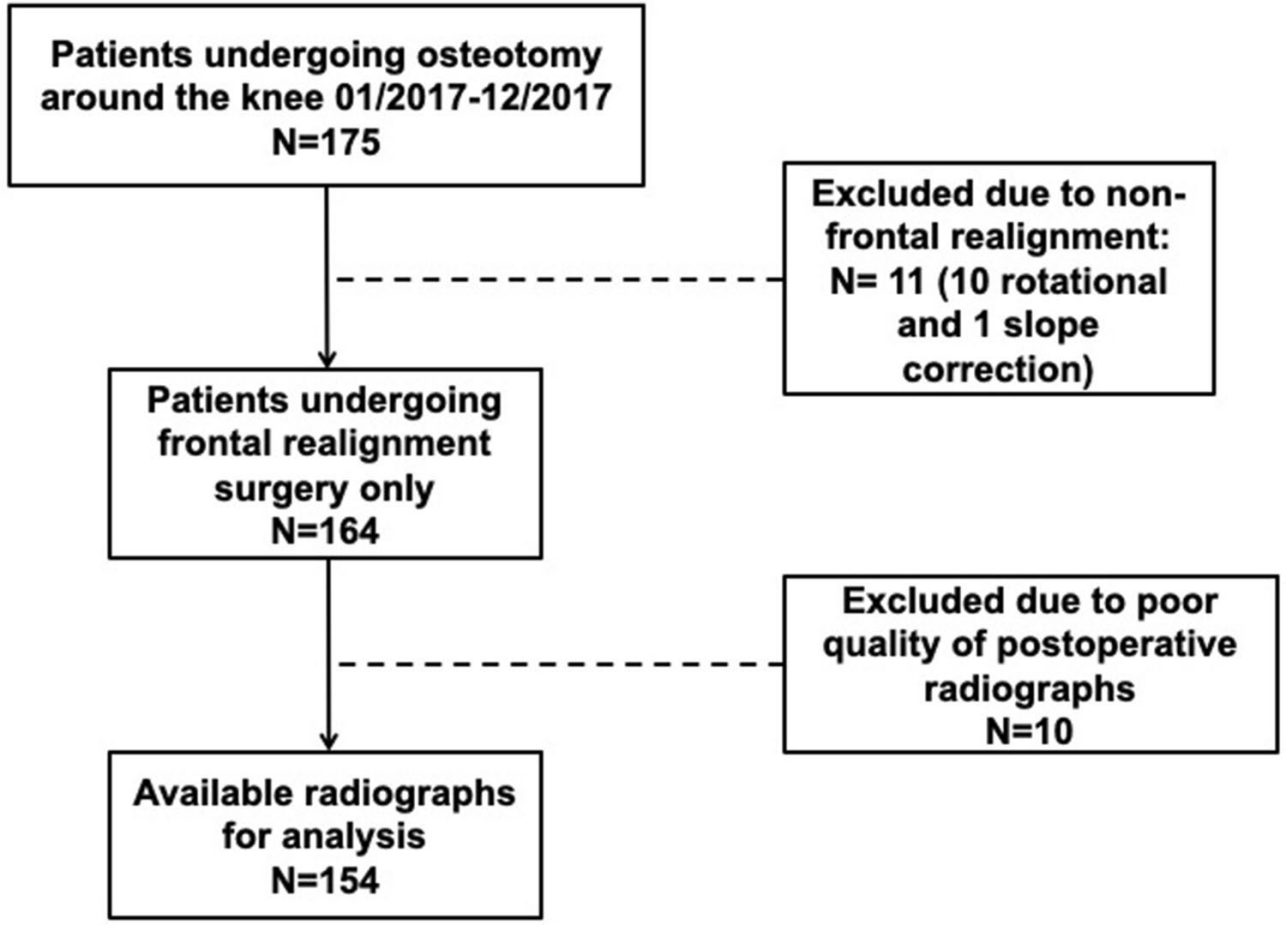
Flowchart illustrating inclusion process

A consecutive series of 154 patients undergoing osteotomies around the knee in the year 2017 were considered eligible for inclusion in the study, provided that pre- and postoperative radiographs were available. Patients were excluded if a multiple plane correction was performed, no magnification device was present on the postoperative radiograph or  if the postoperative image quality was poor. Considering the above criteria, 154 knees of 154 patients undergoing osteotomy were considered eligible for retrospective data retrieval and inclusion in the study (Fig. 1).

### Surgical procedure

All osteotomies were planned using a landmark-based deformity analysis. High tibial osteotomy (HTO) was performed as described by Staubli and Lobenhoffer using a TomoFix™ MHT plate fixator (DePuySynthes, Solothurn, Switzerland) [6, 17, 23]. Distal femoral osteotomy was performed as described by Lobenhoffer [18]. Double level osteotomy was performed as described by Schröter et al. [21]. Seventy HTO procedures, 55 distal femur osteotomies and 29 double level osteotomies were performed. 

### Radiographs

Long-leg weight-bearing radiographs were obtained in accordance to Paley using a 1.3 m cassette (Global Imaging Baltimore, MD). Long-leg antero-posterior standing radiographs were obtained with the patient standing in a bipedal stance in front of the long film cassette. The X-ray tube was positioned 305 cm away. The selected film cassette was of sufficient length to capture the hips knees, and ankles. The magnification with this setup was approximately 5%. A magnification device (250 mm steel ball) was used to calibrate the radiographs. The X-ray beam was centered at the level of the knee joints.

Radiologic technical assistants were instructed to position both legs with the patella centred between the femoral condyles pointing forward. It was of ultimate importance to ensure a standardized radiography. 

Preoperative radiographs were obtained prior to surgery for planning of the deformity correction and were repeated postoperatively after union at the osteotomy site and recovery of limp-free weight bearing.

### Radiographic parameters

**Fig. 2:**
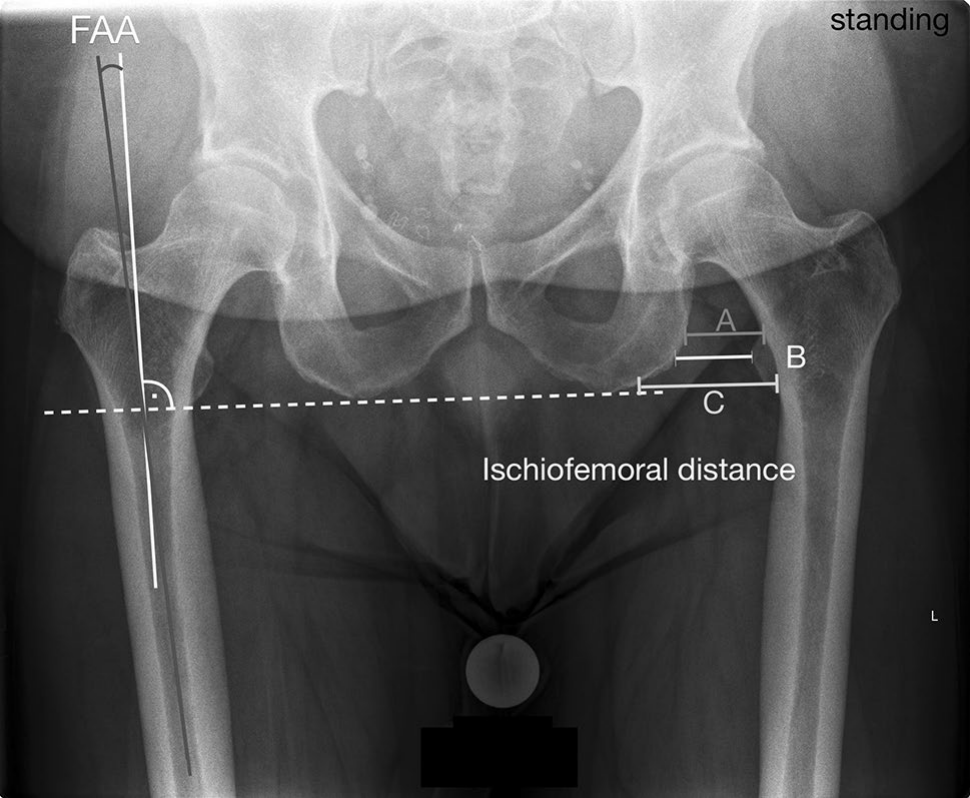
An illustration of the parameters that were particularly defined for this study including the femoral adduction angle (FAA): the angle between the anatomic femoral shaft axis and a line perpendicular to the orientation of the pelvis in the frontal plane (line tangent to the inferior borders of the ischial tuberosities). Frontal ischiofemoral space: the mean of three distances measured between the femur and the ischium at the level of the lesser trochanter and in line with the pelvic orientation. The first distance runs between the lateral cortex of the ischium and the most superior portion of the lesser trochanter (A), the second was parallel to the first between the ischium and the most medial point of the lesser trochanter (B) and the third was parallel to the upper lines running to the most inferior point of the lesser trochanter (C)

Radiographic parameters were determined with an accuracy of 0.1 mm or 0.1° using mediCAD^®^ software (Hectec, Landshut, Germany). The following parameters were measured in accordance with Paley [24]: mechanical medial proximal tibial angle (mMPTA), mechanical lateral distal femoral angle (mLDFA), mechanical lateral proximal femoral angle (mLPFA), joint line convergence angle (JLCA) and the hip knee ankle (HKA) angle. Moreover, two radiographic measures were defined for the specific purpose of this study in order to address the primary research question. The femoral adduction angle (FAA) which was defined as the angle between the anatomic femoral shaft axis and a line perpendicular to the orientation of the pelvis in the frontal plane (line tangent to the inferior borders of the ischial tuberosities) (Fig. 2). The frontal ischiofemoral distance which was defined as the mean of three distances measured between the is ischium and the lesser trochanter in line with the horizontal pelvic orientation. The first line runs between the lateral cortex of the ischium and the most superior portion of the lesser trochanter (A), the second was parallel to the first between the ischium and the most medial point of the lesser trochanter (B) and the third was parallel to the upper lines and ran to the most inferior point of the lesser trochanter (C) (Fig. 2).

### Statistical analysis

Continuous variables were presented as mean ± standard deviation and range. Linear regression was performed to determine the influence of the variety of input variables on the ischiofemoral distance. Comparison between means was performed using the Wilcoxon–Mann–Whitney-Test. A *p* value of < 0.05 was considered statistically significant. SPSS version 24 (IBM, Armonk, New York) was utilized for analysis. Post hoc power analysis showed that a power of 95% was achieved for pre- and post-adduction of the femur as the primary research question, given the sample size of 154 patients and α-value of 5%.

## Results

The examined cohort of patients included 154 knees of 154 patients undergoing osteotomies around the knee due to a bony malalignment and corresponding symptoms. The mean age was 51 ± 11 years. There were 96 male patients and 58 females. All osteotomies performed were around the knee and included 73 high tibial osteotomies (HTO), 54 distal femur osteotomies (DFO) and 27 double level osteotomies. Of all osteotomies, 118 were valgisation osteotomies and 36 were varisation osteotomies. Radiographic findings of knees undergoing varisation and valgisation are illustrated in Tables 1 and 2.

**Table 1:** The radiographic measure in patients undergoing valgisation osteotomy

	Preoperative	Postoperative
HKA° mean ± SD (range)	− 5.8 ± 3 (− 17.0 to 0.4)	0.7 ± 3.0 (− 9.8 to 6.0)
mMPTA° ± SD (range)	86.5 ± 2.6 (78.9–92.4)	90 ± 3.2 (79.4–97.3)
mLDFA ± SD (range)	89.2 ± 2.3 (84.5–99.3)	87.4 ± 2.2 (81.6–97.7)
JLCA ± SD (range)	3.1 ± 2.2 (− 2.1 to 10.3)	2.7 ± 2.1 (− 1.6 to 11.3)
mLPFA ± SD (range)	89.1 ± 5.7 (72.9–106.5)	87.9 ± 5.5 (71.2–101.9)

HKA: Hip Knee Angle (negative represents varus alignment), mMPTA: mechanical medial proximal tibial angle, mLDFA: mechanical laterale distal femoral angle, JLCA: joint line congruency angle, mLPFA: mechanical proximal femur angle, SD: Standard deviation

**Table 2:** Presenting the radiographic measure in patients undergoing varisation osteotomy

	Preoperative	Postoperative
HKA° mean ± SD (range)	5.4 ± 2.9 (1.1–11.5)	− 0.7 ± 2.0 (− 4.2 to 3.6)
mMPTA° ± SD (range)	90.8 ± 3.0 (85.3–97.8)	88.8 ± 3.0 (82.9–96.6)
mLDFA ± SD (range)	85.4 ± 2.4 (78.3–90.7)	89.5 ± 2.5 (84.4–94.2)
JLCA ± SD (range)	0 ± 2.2 (− 3.8 to 7.7)	0 ± 2.9 (− 6 to 10.1)
mLPFA ± SD (range)	86.7 ± 7.2 (70.5–103.0)	87.1 ± 7.0 (70.7–103.3)

*HKA* Hip Knee Angle (negative represents varus alignment), *mMPTA* mechanical medial proximal tibial angle, *mLDFA* mechanical laterale distal femoral angle, *JLCA* joint line congruency angle, *mLPFA* mechanical proximal femur angle, *SD* Standard deviation

**Fig. 3:**
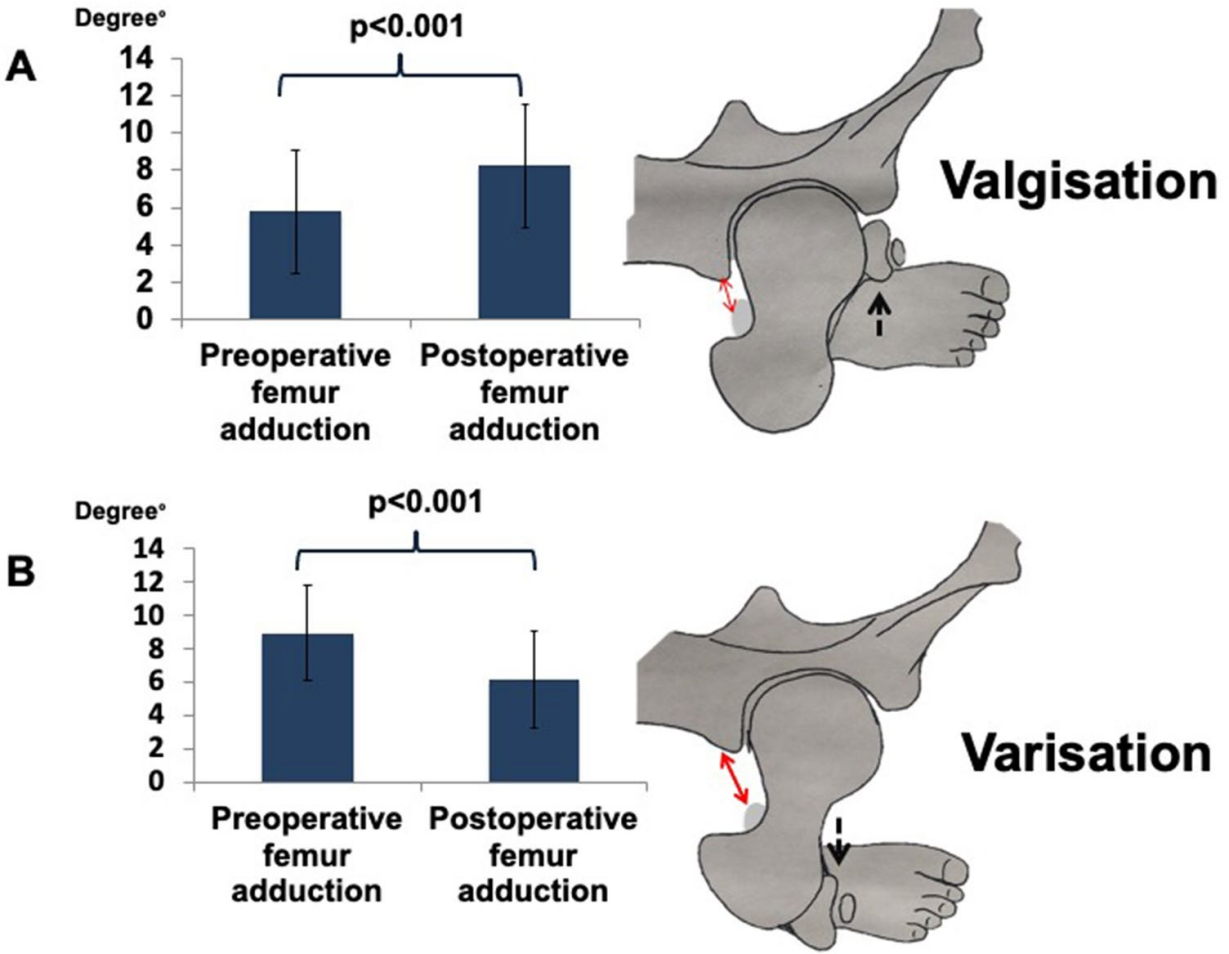
Influence of an osteotomy around the knee on the ischiofemoral space. **a** The change in ischiofemoral space after a valgisation osteotomy. **b** The change in ischiofemoral space after varisation. The arrows represent the ischiofemoral space

The results show that a valgisation osteotomy around the knee was associated with an increase in the adduction of the femoral shaft in relation to the pelvis (*p* < 0.001), as demonstrated by the FAA angle. Figure 3a provides an illustration of the increase in adduction after valgisation osteotomy. On the other hand, a varisation osteotomy around the knee was associated with a decrease in FAA (*p* < 0.001) and therefore induced abduction of the femur. Figure 3b provides an illustration of the increase in abduction after varisation osteotomy.

**Fig. 4:**
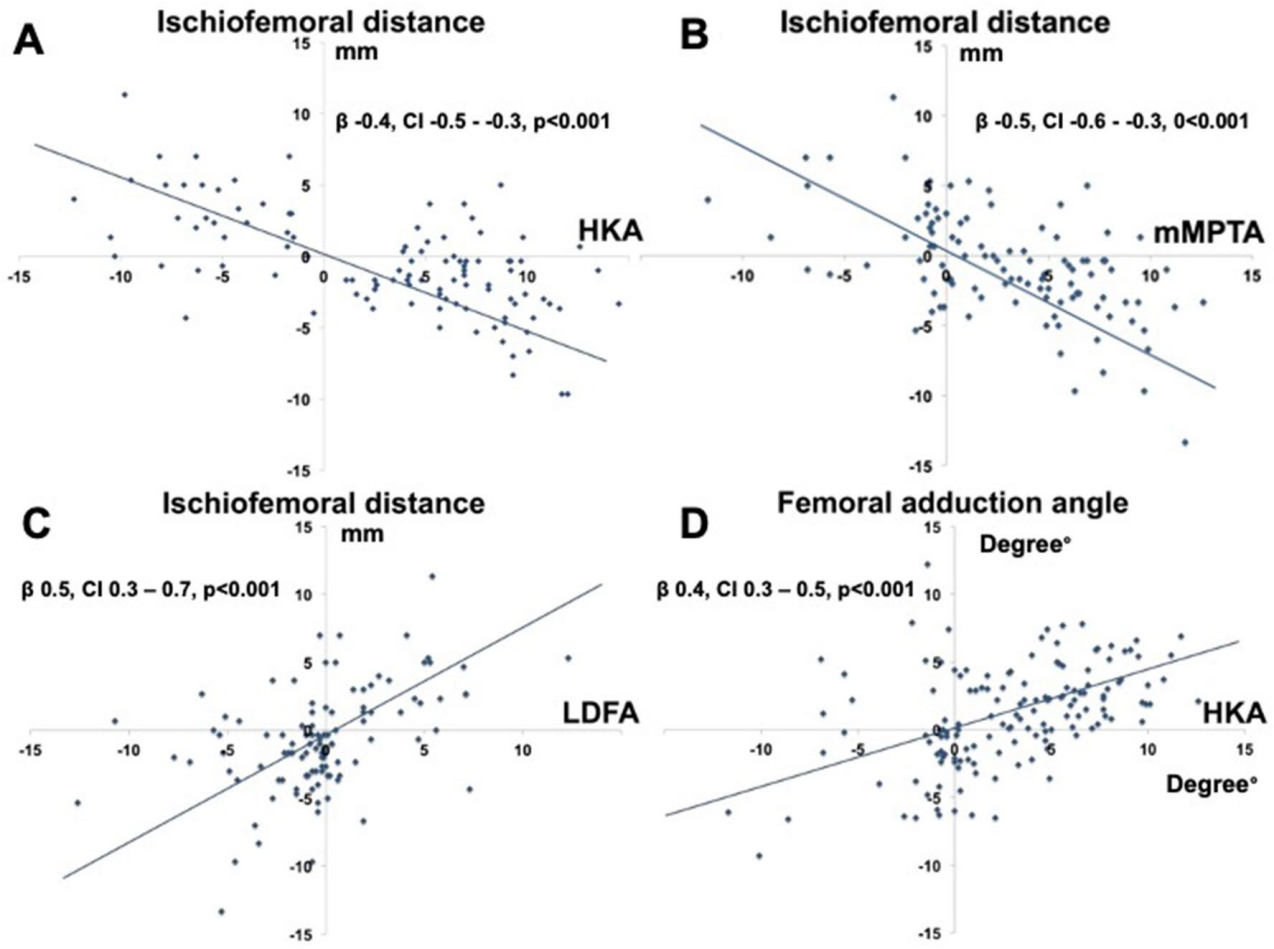
Linear regression analysis. **a** Influence of the change in hip knee angle (HKA) on the ischiofemoral distance. **b** Influence of the mechanical medial proximal tibial angle (mMPTA) on the ischiofemoral distance. **c** Influence of the lateral distal femur angle on the ischiofemoral distance. **d** Influence of HKA on the femoral adduction angle (FAA)

Regression analysis demonstrated an association between the alignment of the knee (HKA angle) and the ischiofemoral distance (β − 0.4, Confidence Interval (CI) − 0.5 to − 0.3, *p* < 0.001). A more valgus frontal knee alignment was associated with a reduced ischiofemoral distance (Fig. 4a).

This effect was demonstrated on both tibial and femoral metaphyseal levels. An increase in valgus orientation of the proximal metaphysis of the tibia was associated with a decrease in the ischiofemoral distance (β − 0.5, CI − 0.6 to − 0.3, 0 < 0.001) (Fig. 4b). An increase in the valgus orientation of the distal femoral metaphysis was also associated with a decrease in ischiofemoral distance (β 0.5, CI 0.3–0.7, *p* < 0.001) (Fig. 4c).

It was also shown that a more valgus overall frontal knee alignment (HKA angle) induced adduction of the femur in relation to the pelvis in an upright standing position (β 0.4, CI 0.3–0.5, *p* < 0.001) (Fig. 4d) The increase in FAA was further shown to correlate with a reduction in ischiofemoral distance based on linear regression (β 0.5, CI 0.1–0.8, *p* = 0.026).

## Discussion

This study demonstrated an association between the frontal knee alignment and the ischiofemoral space of the hip joint. Realignment surgery around the knee has shown a significant influence on the ipsilateral ischiofemoral space regardless of the site of the osteotomy. Valgisation osteotomy resulted in a decrease in the ischiofemoral space, varisation osteotomy in an increase.

The novelty aspect of this study is underlined by consideration of the frontal knee alignment in the overall concept of extra-articular hip impingement. The introduction of this component into the overall hip workup could optimize individual treatment planning and ultimately widen the spectrum of therapeutic treatment options.

Ischiofemoral impingement is increasingly being recognized for its role in the pathomechanism of atypical hip pain and particularly in conjunction with posterior hip impingement [7, 16, 22]. Several pathomorphological features have been linked to this entity, including proximal femur deformities in Legg–Calve–Perthes disease or in hips with coxa valga and high femoral antetorsion [19, 22]. In these patients, the orientation of the femoral neck is more posterior and closer to the pelvis in a normal standing position and an extra-articular ischiofemoral conflict is more likely to occur due to the reduced ischiofemoral space [11, 22, 27]. However, considering the results of this study, it is fair to state that regardless of femoral torsion and the orientation of the femoral neck in relation to the pelvis, the frontal alignment of the knee is also a determinant of the ischiofemoral space.

Therefore, it is fair to underline the need for appreciating the knee alignment in the overall workup of a symptomatic hip. Realignment surgery around the knee may resolve a bony conflict in the hip. Especially, if the obliterated ischiofemoral space is resultant to a valgus knee alignment. A concept that must be understood.

The concept is also relevant to the knee surgeon in the overall workup of the painful knee and when planning knee alignment correction. It is of upmost importance to appreciate the influence of the correction on the adjacent hip joint to prevent the occurrence of an unwanted effect. Given that pain disorders of the ankle have also been reported as a result of realignment of the knee, it can only be underlined that there is a true need of deepening the understanding of limb realignment with particular emphasis on further developing planning tools [1, 5, 10].

**Fig. 5:**
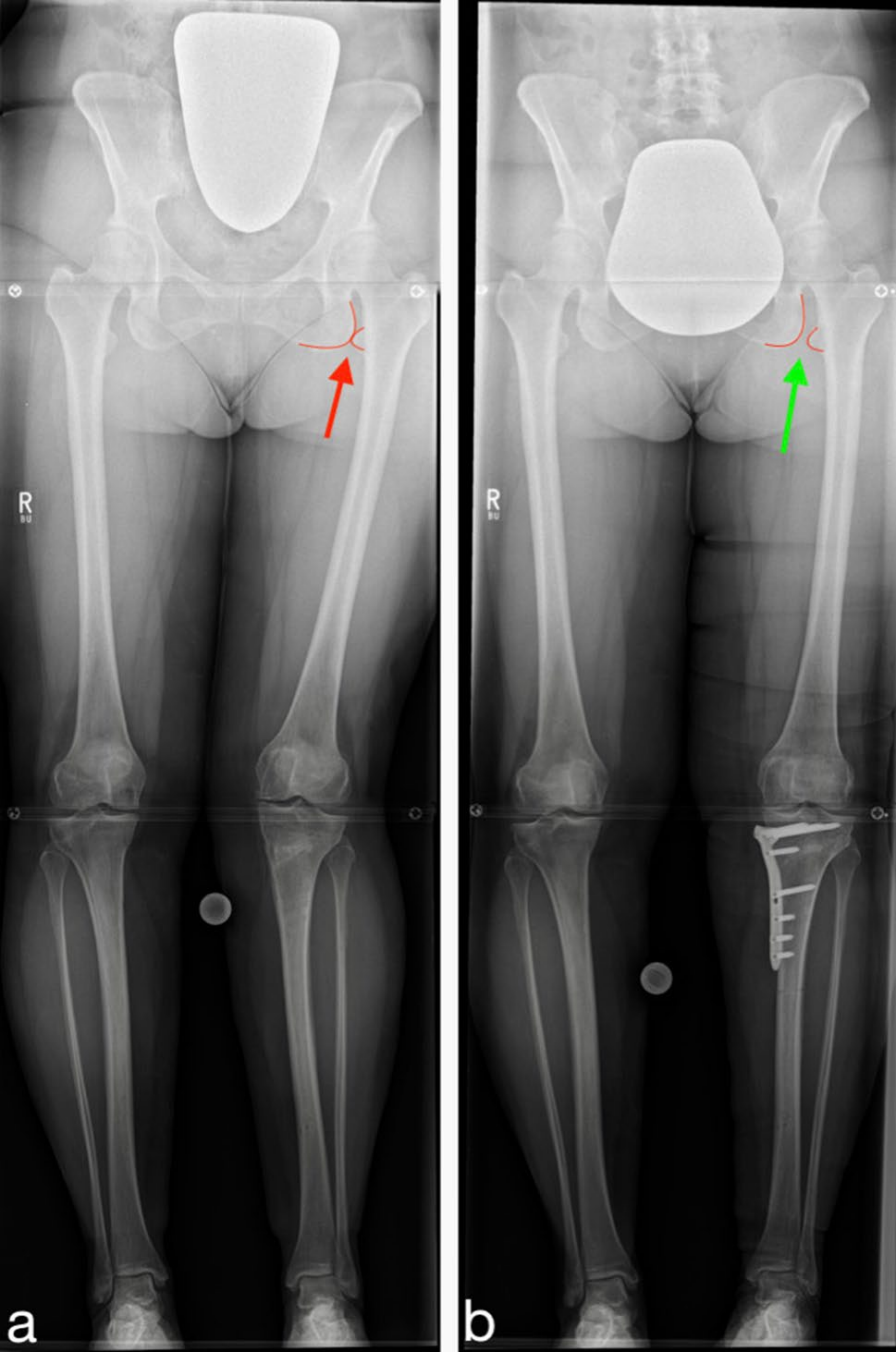
Radiographic demonstration of a patient who presented with new-onset symptomatic disabling hip pain on the left side after having undergone a high tibial valgisation osteotomy on due to medial knee gonalgia. **a** Over-correction with reverse obliquity of the joint line and a merely obliterated ischiofemoral space (arrow). **b** The situation after a closing wedge high tibial osteotomy and restoration of the ischiofemoral space 6 months postoperatively, the patient was pain free

Figure 5 represents an example of the above concept in a patient who was referred to the outpatient clinic with severe hip pain that has occured after having undergone a high tibial osteotomy for the treatment of medial knee gonalgia. The knee pain was no longer present, though the new-onset hip pain caused extreme disability to the extend that walking was no longer possible without crutches. It was obvious from the radiographs that the ischiofemoral space was obliterated due to  excessive valgisation of the knee. A revision procedure was necessary to reverse the effect of the osteotomy (Fig. 5). After re-establishing the ischiofemoral distance, the patient was pain-free. This example demonstrates that the consequence of overcorrection of a varus knee whilst neglecting the hip may be devastating.

The plausible explanation can be seen in the idea that after a knee valgisation osteotomy, adduction of the femur would have to occur to maintain contact between the foot and the floor, this would ultimately result in the reduction or obliteration of the ischiofemoral distance. In patients with a naturally narrow ischiofemoral space, impingement may occur (Fig. 5).

The limitations of this study could be seen in the fact that femoral torsion was not determined and included as an input variable. Despite the valuable input, this measure would have resulted in radiation exposure violating ethical standards. To account for that problem, patient who underwent osteotomies around the knee were considered to control for most variables including torsion. Furthermore, a three-dimensional determination of the ischiofemoral space would provide a more accurate depiction. This would mandate complex imaging in a functional standing position. The same applies for dynamic testing. Given that the aim of this study was to prove the concept, the authors agreed on the sufficiency of the design chosen in this study.

## Conclusion

The results of this study highlight the influence of the frontal knee alignment on the ischiofemoral space of the hip. Correction of a malalignment of the knee may resolve an ischiofemoral conflict in the hip. The concept deserves inclusion in the diagnostic workup of both the hip and knee joints.
